# Understanding and mitigating cancer-related financial toxicity in China: challenges and recommendations

**DOI:** 10.1016/j.lanwpc.2025.101601

**Published:** 2025-06-17

**Authors:** Weijie Xing, Zhenqi Lu, Yuhan Lu, Huiying Qin, Juan Xie, Qiongyao Guan

**Affiliations:** a School of Nursing, Fudan Universtiy, Shanghai, China; b Department of Nursing, Fudan University Shanghai Cancer Center, Shanghai, China; c Key Laboratory of Carcinogenesis and Translational Research (Ministry of Education/Beijing), Peking University Cancer Hospital & Institute, Beijing, China; d State Key Laboratory of Oncology in South China, Collaborative Innovation Center for Cancer Medicine, Sun Yat-sen University Cancer Center, Guangzhou, China; e Department of Nursing, Shaanxi Provincial Cancer Hospital, Xi'an, China; f Department of Nursing, The Third Affiliated Hospital of Kunming Medical University/Yunnan Cancer Hospital, Kunming, China

Cancer imposes a substantial financial burden on patients worldwide, and China is no exception. As the country faces a rising cancer incidence and improving survival rates, financial toxicity—the objective financial burden and subjective distress caused by the cost of cancer treatment and care—has emerged as a critical but under-addressed issue in oncology. China's unique healthcare, insurance, and cultural context presents challenges to financial toxicity that differ from those seen elsewhere in the world. In this Comment, we highlight the current status, challenges, and future directions for addressing financial toxicity among cancer patients in China.

## Financial toxicity in China

### The prevalence of financial toxicity in China

A nationwide cross-sectional study involved 1208 cancer patients from 12 hospitals across three economically diverse provinces revealed a strikingly high FT prevalence of 82.6%, with 40.9% experiencing severe FT.^1^ The prevalence exceeded the 51% reported in a meta-analysis that pooled data from 16 studies primarily conducted in developed countries,^2^ likely due to variations in healthcare systems, economic levels, and cultural backgrounds between developed and developing countries. Given China's vast geographic span and substantial regional economic disparities, the financial burden of cancer care varies considerably across different areas. Patients from middle- and low-income regions had significantly more severe FT than their high-income counterparts, consistent with global research trends and highlighting the need for context-specific interventions to enhance financial resilience in under-resources areas.^3^ Moreover, a multi-center longitudinal study further demonstrated that the burden of FT is most pronounced during the early survivorship phase—particularly three months post-surgery, indicating that the initial post-treatment period represents a critical window for implementing targeted interventions to mitigating long-term FT.^4^

### The components of financial toxicity in China

**Fig. 1:**
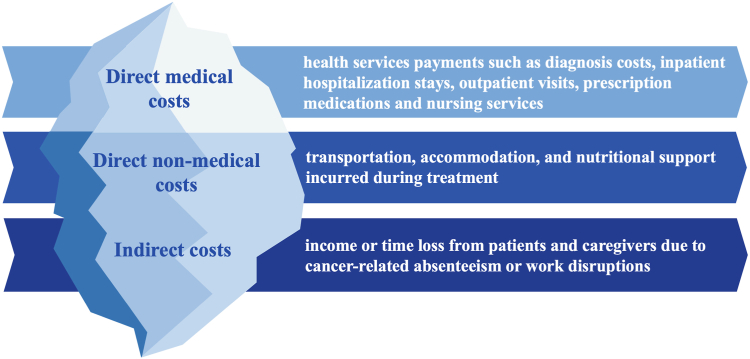
The components of financial toxicity in China.

The components of cancer-related FT in China can be classified into three categories (Fig. 1): (1) Direct medical costs, such as out-of-pocket (OOP) health services payments such as diagnosis costs, inpatient hospitalization stays, outpatient visits, prescription medications and nursing services; (2) Direct non-medical costs, including transportation, accommodation, and nutritional support incurred during treatment; and (3) Indirect costs, such as income or time loss from patients and caregivers due to cancer-related work disruptions. A cross-sectional study which included 13,745 patients diagnosed with six leading cancers from 84 hospitals across 17 provinces in China reported the comprehensive costs of cancer borne by patients.^5^ During the treatment phase, direct medical costs ranged from $4685 to $12,024, direct non-medical costs ranged from $402 to $1040, and indirect costs ranged from $1273 to $2846. During the follow-up phase, direct medical costs ranged from $4170 to $8276, direct non-medical costs ranged from $378 to $760, and indirect costs ranged from $1113 to $3002. While direct medical costs account for the largest portion of total expenses, non-medical and indirect costs also significantly contribute to financial stress and are often overlooked in traditional assessments.

### The risk factors and consequences of financial toxicity in China

A systematic review of financial toxicity among cancer patients in China showed that higher levels of FT were observed among patients with advanced cancer stage, longer hospital stays, combination therapy, more treatment-related side effects, lower educational level, lower household income, unemployed status, rural residence, lack of private medical insurance, poor informational and emotional support and inadequate cost communication.^6^ The most common consequences of FT were reduced health-related quality of life and treatment nonadherence.^6^

## Challenges and gaps

### Cross-regional healthcare-seeking increases non-medical costs

In China, disparities in the distribution of medical resources between urban and rural areas often leads to patients travelling long distances to access high-quality treatment.^7^ These cross-regional journeys incur substantial indirect costs, including transportation, lodging, and nutritional support. In addition, family caregivers frequently experience work disruption or income loss, further intensifying the financial burden. Such non-medical expenses are rarely covered by any insurance schemes, yet they significantly contribute to the patients’ overall financial stress.

### Limited insurance diversity weakens financial risk protection

China's cancer care system is predominantly supported by the Basic Medical Insurance (BMI) scheme, which covers approximately 95% of the population.^8^ However, reimbursement levels vary by employment status: employed individuals typically receive higher coverage, while non-working groups—such as children, the elderly, and rural residents—face lower reimbursement rates. Furthermore, BMI also does not fully cover the costs of innovative therapies or advanced diagnostic technologies, leaving patients exposed to substantial out-of-pocket spending. Although public-private insurance models are being explored, commercial health insurance remains underutilized, accounting for only 3.8% of total health expenditure, limiting patients' financial protect during prolonged cancer treatment.^9^

### Insufficient cost communication undermines informed treatment decisions

In China, patients commonly defer medical decisions to physicians, leading to limited engagement in treatment planning and insufficient discussion of care-related costs.^10^ This communication gap not only hinders patients' understanding of the financial implications of their choices but also contributes to a lack of awareness that cancer often requires long-term, ongoing treatment. This lack of awareness leads to poor anticipation and planning for future medical expenses, weakening patients’ ability to cope with financial toxicity and potentially compromising the sustainability and affordability of their treatment choices.

### Family-centered cultural norms intensify household financial vulnerability

In China, strong family-oriented cultural norms often motivate relatives to support cancer treatment at all costs, sometimes resulting in significant depletion of household resources.^11^ While this commitment reflects deep emotional bonds and a sense of familial duty, it may inadvertently accelerate financial hardship. Furthermore, cancer is frequently perceived as a life-threatening or incurable disease, leading many families to adopt a “total rest” approach that discourages patients from engaging in any work or household responsibilities. Although intended to foster recovery, such practices may delay functional rehabilitation and further erode the household's economic resilience.

## Recommendations for future directions

**Fig. 2:**
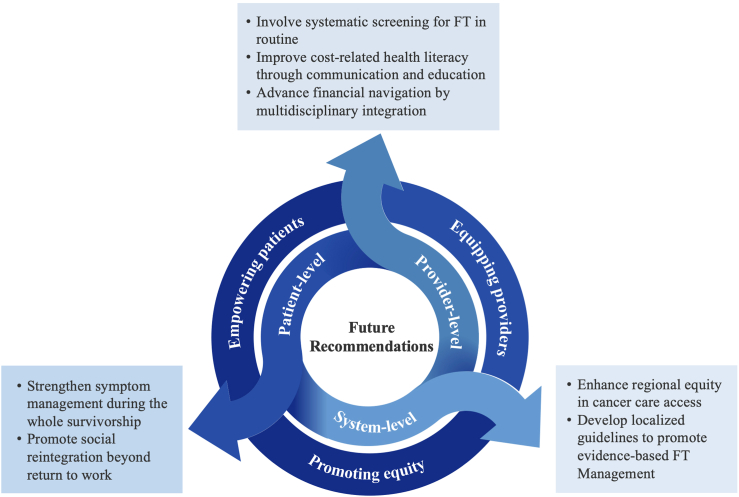
Recommendations for future directions.

To address the multifaceted nature of FT, we propose a series of actionable recommendations spanning three levels—the patient level, the provider level, and the system level (Fig. 2).

### Patient-level recommendations

#### Strengthening symptom management during the whole survivorship

Multiple studies have demonstrated the link between symptom burden and financial toxicity.^12^ Uncontrolled symptoms often lead to additional healthcare utilization, thereby increasing financial strain. Conversely, severe financial distress hinders access to effective symptom management.^13^ Proactive symptom monitoring and management throughout the survivorship phase is essential to breaking this vicious cycle-both alleviating patients’ symptoms and reducing their financial distress.

#### Promoting social reintegration beyond return to work

Cancer survivors' return to work not only restores their economic productivity but also alleviates long-term financial toxicity.^14^ For survivors who are unable to resume formal employment or are beyond working age, engaging in unpaid productive roles—such as taking on family responsibilities—can also help ease the household's financial burden.^15^ Future efforts should explore the dynamic transition of cancer survivors from social withdrawal to reintegration, and develop multilayered strategies that empower them to reclaim their roles as vital contributors to family and society, thereby mitigating the long-term economic impact of cancer.

### Provider-level recommendations

#### Involving systematic screening for financial toxicity in routine

Patients’ perceived financial toxicity varies widely and follows different trajectories over time.^16^ While physical and psychological symptom assessments are well integrated into oncology nursing practice, evaluations of financial burden remain superficial. Incorporating concise and validated tools for routine screening can help assess both current financial distress and the risk of developing severe financial toxicity, enabling early identification and targeted intervention for high-risk patients.^17^

#### Improving cost-related health literacy through communication and education

Enhancing cost-related health literacy among cancer patients helps improve their understanding of treatment costs, boosts their confidence in financial management, and supports more informed and reasonable medical decisions.^18^ Healthcare providers should initiate discussions about treatment cost early, helping patients consider both clinical benefit and financial affordability. Tailored education should also be provided to improve patients’ awareness of insurance coverage, reimbursement policies, and available financial assistance, thereby empowering them to better cope with financial stress.

#### Advancing financial navigation by multidisciplinary integration

Among available interventions, financial navigation is the most frequently used approach for alleviating financial toxicity.^19^ Evidence from both international and local studies has confirmed its effectiveness by providing personalized, timely support—helping patients navigate insurance policies, estimate treatment costs, access financial aid, and develop practical expense management strategies.^20^^,^^21^ In the future, it is expected to develop a multidisciplinary model integrating healthcare providers, social workers, financial counselors and digital tools to enhance the sustainability of interventions.

### System-level recommendations

#### Enhancing regional equity in cancer care access

Promoting regional equity in cancer care requires national-level policy efforts. However, at the institutional level, concrete actions can be taken. Healthcare providers can leverage internet hospital platforms to offer remote consultations, follow-ups, and symptom management, reducing the need for survivors to travel long distances.^22^ Additionally, medical consortiums facilitate the sharing of treatment protocols, clinical experiences, and nursing practices across institutions, enhancing local capacity.^23^ These approaches allow cancer survivors to access high-quality, continuous care closer to home, easing indirect costs and advancing equitable service delivery.

#### Developing localized guidelines to promote evidence-based FT management

While a growing number of Chinese research teams have made significant progress to address cancer-related financial toxicity, their impact remains geographically and institutionally limited. Although existing international consensus statements offer valuable reference, differences in economic conditions, healthcare systems and cultural norms limit the direct applicability of international consensus in China. There is an urgent need to develop localized guidelines or consensus tailored to China's specific clinical context, thereby supporting the implementation of evidence-based practices.

Financial toxicity is a globally shared challenge among cancer patients, yet its manifestations and solutions are deeply shaped by local contexts. This Comment highlights the specific challenges of financial toxicity in China and proposes recommendations to guide practice. These insights can inform future strategies in China and other settings with similar health system and socioeconomic contexts.

## Contributors

XW-conceptualisation, funding acquisition, methodology, writing-original draft. LZ-concepualisation, investigation, writing-review & editing. All other authors contributed to writing-review & editing.

## Declaration of interests

XW was funded by the National Natural Science Foundation of China (72004034) and China Medical Board (20-371).
